# Ontology and Function of Fibroblast-Like and Macrophage-Like Synoviocytes: How Do They Talk to Each Other and Can They Be Targeted for Rheumatoid Arthritis Therapy?

**DOI:** 10.3389/fimmu.2018.01467

**Published:** 2018-06-26

**Authors:** Jiajie Tu, Wenming Hong, Pengying Zhang, Xinming Wang, Heinrich Körner, Wei Wei

**Affiliations:** ^1^Key Laboratory of Anti-Inflammatory and Immune Medicine, Ministry of Education, Anhui Collaborative Innovation Center of Anti-Inflammatory and Immune Medicine, Institute of Clinical Pharmacology, Anhui Medical University, Hefei, China; ^2^First Affiliated Hospital of Anhui Medical University, Hefei, China

**Keywords:** fibroblast-like synoviocytes, macrophage-like synoviocytes, rheumatoid arthritis, synovium, ontology, treatment

## Abstract

Fibroblast-like synoviocytes (FLS) and macrophage-like synoviocytes (MLS) are the two main cellular components of the synovium. It has been widely reported that FLS and MLS play essential roles in the joint pathology of rheumatoid arthritis (RA). Although various studies have analyzed both human and animal tissues and have shown that both cell types are involved in different stages of RA, ontology, and specific functions of both cell populations and their interactions are not well understood. In this review, we will summarize recent research on FLS and MLS in RA and focus on the development and function of two predominant synovial cell types. In addition, we will discuss the communication between FLS or MLS and highlight potential treatments for RA that involve synoviocytes.

## Ontogenesis of Macrophage-Like Synoviocytes (MLS) and Fibroblast-Like Synoviocytes (FLS)

The lining of the synovium inside the fibrous outer layer (subintima) consists of two to three layers of cells. This intimal synovial lining (intima) is composed predominantly of two cell types: MLS (or type A synoviocytes) and FLS (or type B synoviocytes) (1).

Fibroblast-like synoviocytes are more abundant than MLS and constitute the central cellular component of the intima (2). The ontogeny of FLS is unclear, although it has been reported that FLS descent from mesenchymal stem cells and display some typical fibroblast markers, including the surface marker Thy-1 (CD90), integrins such as ICAM1, and the extracellular matrix proteins type IV and V collagens (2). The notion of a mesenchymal origin was supported by linage-tracing of Gdf5^+^ mesenchymal stromal/stem cells in the synovial tissue. These experiments showed a potential contribution of these stem cells to synovial homeostasis (3). Furthermore, in addition to various general fibroblast markers, such as vimentin and α-smooth muscle actin, some specific FLS markers have been identified such as the enzyme UDP-glucose 6-dehydrogenase (4), vascular cell adhesion molecule-1, and cadherin-11 (CDH11) (5).

The ontogeny of the second synovial cell type MLS has been elucidated to some extent in recent years. Tissue-resident, phagocytic cells were identified more than a century ago by Elie Metchnikoff and described in considerable detail by Cohn and co-workers (4, 6). Originally, researchers held the opinion that these tissue-resident macrophages originated in the bone marrow and reached their specific tissue *via* peripheral circulation (7). This concept was challenged following the discovery of fate mapping technology that made it possible to follow macrophage development from precursor to mature cells. Consequently, the origins of macrophages in different tissues were redefined (8–19). The general principle that was developed in this body of work is that macrophages from different organs/tissues were derived from embryonic precursor cells and maintained by independent, slow proliferation (12). First, a fate mapping strategy which was employed during the prenatal and perinatal period proved that microglial cells in the CNS were derived from the yolk sac at an early embryonic stage (8). Similar approaches were used to analyze the ontogeny of various other tissue-resident macrophages. Macrophages in the epidermis (15) and pancreas (17) were shown to be derived from hemopoietic precursor cells originating from both yolk sac and fetal liver, while macrophages in the dermis are exclusively derived from fetal liver precursor cells (18). In most solid tissue, organs such as liver, kidney, lung, and spleen macrophages are of mixed origin from fetal liver and from monocytes that enter the tissue from circulation after birth (9, 11, 12, 20). The monocytic infiltration into the tissue with subsequent differentiation to macrophages can also support homeostasis but is in most cases limited to an ongoing inflammatory response (21).

**Figure 1:**
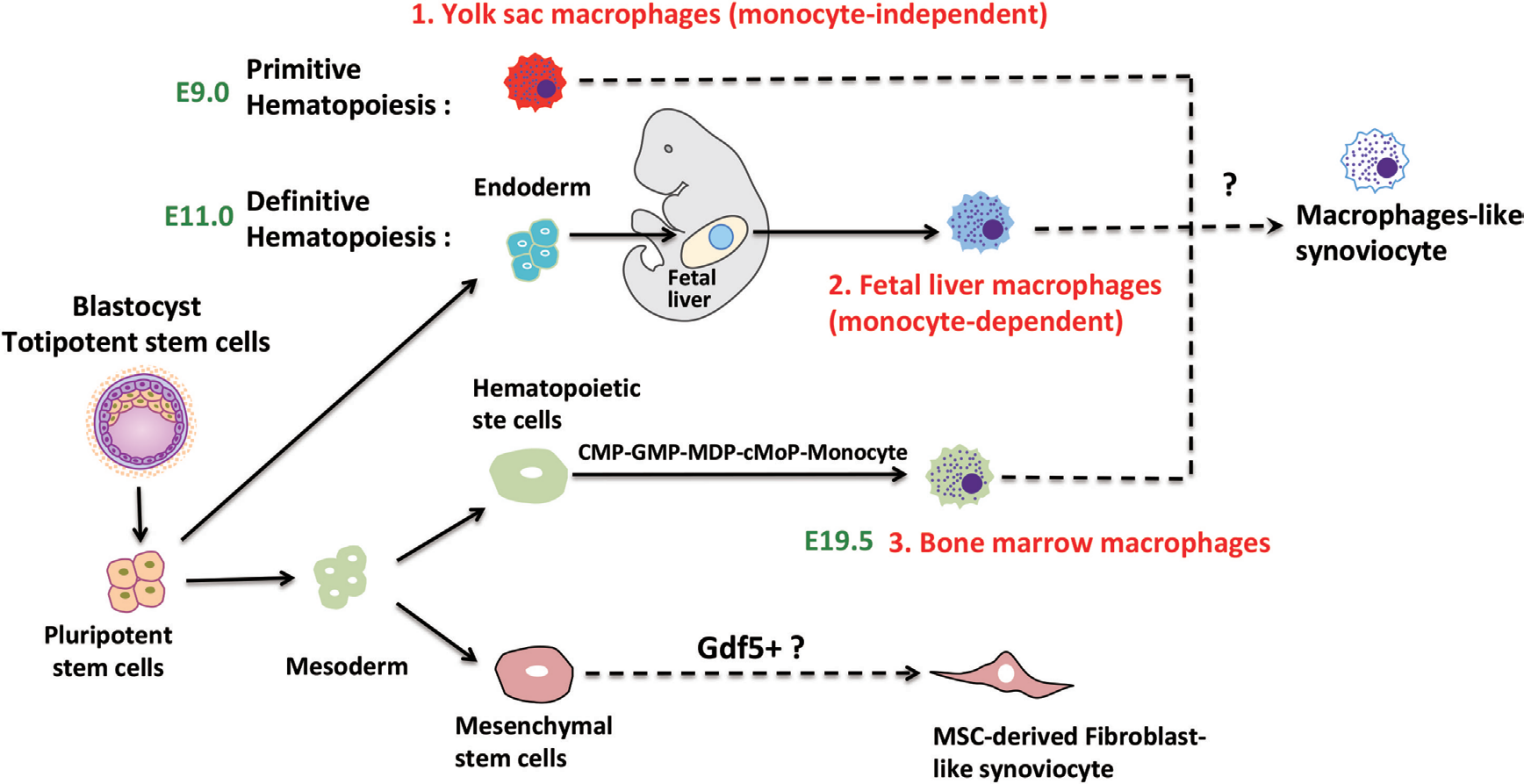
Speculative ontogeny of fibroblast-like synoviocytes (FLS) and macrophage-like synoviocytes (MLS). Macrophages from different organs/tissues are derived from embryonic stem cells (primitive and definitive hematopoiesis) or circulating monocytes (22). During murine embryogenesis, primitive hematopoiesis is firstly detected in blood islands of the yolk sac at around E7.5, which followed by definitive hematopoiesis in aorta-gonad-mesonephros (AGM) regions, then shifts to the fetal liver, spleen, and bone marrow. MLS most certainly are derived from embryonic precursor cells but the detailed ontogeny is still elusive. FLS may originate from Gdf5 + mesenchymal cells (E7.5, Day 7.5 at embryonic stage; E9.0, Day 9 at embryonic stage; E11.0, Day 11 at embryonic stage; E19.5, Day 19.5 at embryonic stage).

A major problem for the identification of individual monocyte/macrophage populations is the redundancy of marker molecules. Generally, murine tissue-resident macrophages which are derived from embryonic precursor cells are F4/80^high^. On the other hand, bone marrow-derived monocytes/macrophages display the specific markers chemokine receptor type 2 (CCR2) and Ly6C but are also F4/80^intermediate^. The ontogeny of macrophages is summarized in Figure 1 and has been discussed in more detail elsewhere (22).

A transcriptome profiling of FLS and MLS isolated from rheumatoid arthritis (RA) patients confirmed that MLS are macrophages and have strong inflammatory tendencies. Interestingly, it also showed that FLS were able to substantially contribute to the inflammatory response (23). However, despite the progress in determining the development of tissue-resident macrophages in general, the origin of MLS is still elusive. A recent report that focused on the role of recruited monocytes in the synovium by using a serum-induced arthritis mouse model (24) indicated that MLS were derived from both embryonic precursor cells and the bone marrow. The current knowledge about the specific origin of MLS from different sources in RA will be discussed below.

## The Roles of FLS and MLS in RA

### FLS in RA

**Figure 2:**
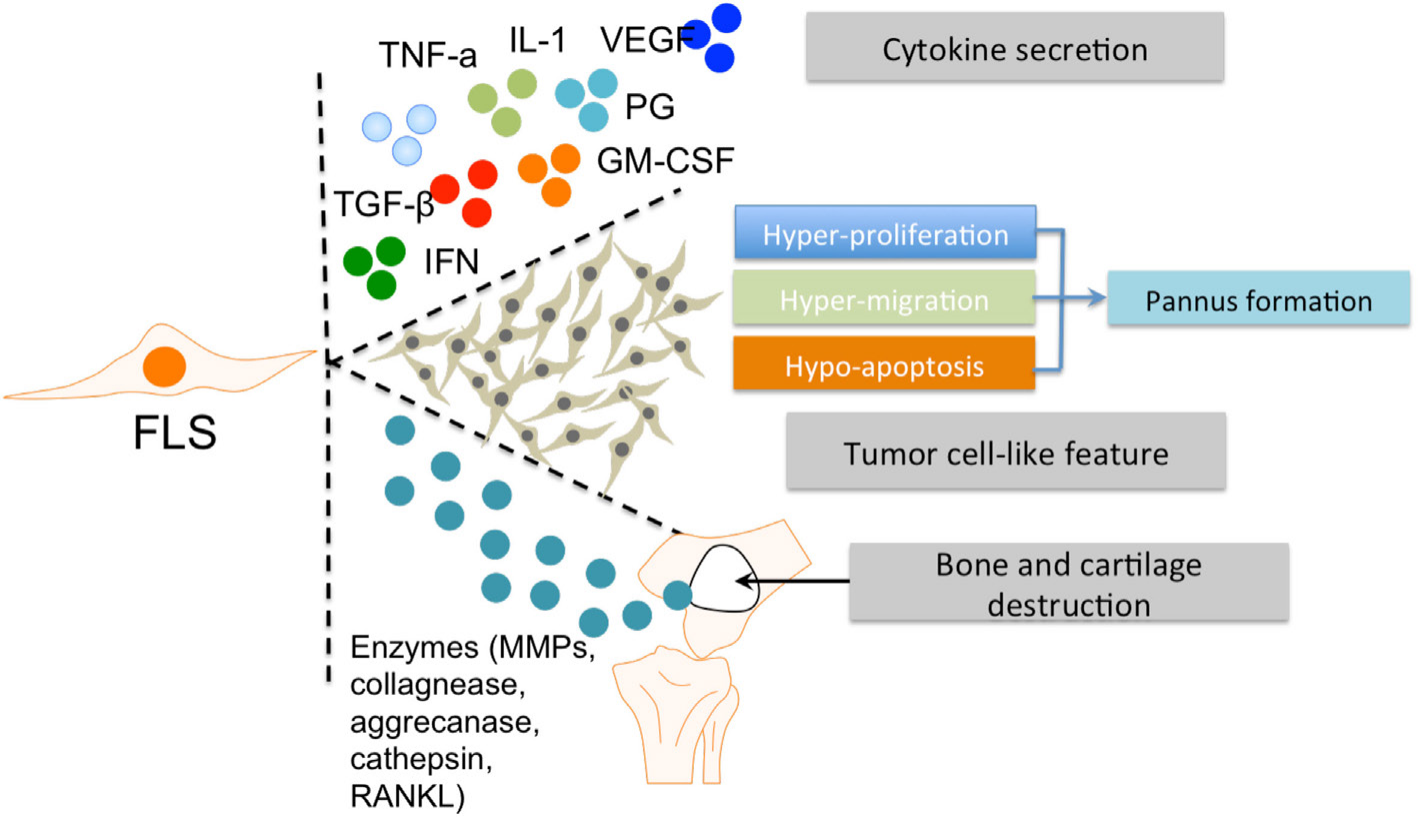
The roles of FLS in RA. FLS are involved in many pathological aspects of RA by promoting synovitis, pannus growth, and cartilage/bone destruction. Abbreviations: FLS, fibroblast-like synoviocytes; TNF-α, tumor necrosis factor α; IL-1, interleukin 1; VEGF, vascular endothelial growth factor; TGF-β, transforming growth factor β; PG, prostaglandin; IFN, interferon; GM-CSF, granulocyte-macrophage colony-stimulating factor; MMPs, matrix metalloproteinases; RANKL, receptor activator of nuclear factor kappa-B ligand; RA, rheumatoid arthritis.

The role of FLS in RA has been well established (2). FLS are involved in many pathological aspects of RA by promoting synovitis, pannus growth, and ultimately, cartilage/bone destruction (Figure 2).

In rheumatoid inflammation, FLS secrete various pro-inflammatory factors. The main cytokine secreted by FLS is interleukin (IL)-6 (25), which is induced by IL-1 and tumor necrosis factor (TNF)-α (26, 27). Another essential pro-inflammatory factor is granulocyte-macrophage colony-stimulating factor (GM-CSF) (28, 29). Cytokine secretions from FLS can influence the interaction of FLS and other immune cells, especially MLS, with synovium (30). In addition, these abovementioned cytokines can activate M1 polarization of MLS (31).

Fibroblast-like synoviocytes also produce a panel of anti-inflammatory factors (including prostaglandins and vascular endothelial growth factor) or factors with a combination of pro-inflammatory and anti-inflammatory effects under inflammatory conditions. For example, transforming growth factor β secreted by FLS has a dual effect on MLS in RA, depending on concentration and exposure time (32, 33). Other factors, such as Type 1 interferons induce similar mixed phenotypes in RA (34, 35). Therefore, FLS have the ability to drive the pro-inflammatory differentiation of MLS by producing various cytokines within the RA synovial microenvironment. The observation that FLS are able to produce various cytokines *in vitro*, even in the absence of exogenous stimuli (1) indicates that both intrinsic signaling and external communication (e.g., with MLS) synergistically form the pro-inflammatory profile of FLS in RA. This is supported by the results of a profiling of the transcriptome of both cell types (23).

Fibroblast-like synoviocytes hyperplasia in the inflamed synovium is a hallmark of RA. This increased presence of FLS is due to strong proliferation and reduced apoptosis (36, 37). The capacity of FLS for hyperproliferation and abnormal survival consequently promote pannus formation within the synovium (38). The hypersurvival character of FLS in RA is considered tumor-like behavior (39).

After activation by other cellular components in the pathological RA synovium (including MLS), FLS produce several enzymes that are important for their invasive features and contribute to tissue destruction. Matrix metalloproteinases are known to cause degradation of the extracellular matrix. Other factors secreted by FLS include collagenases, aggrecanases, cathepsins, and RANKL, which play an important role in the invasive activities of FLS and subsequent bone and cartilage destruction (14, 15). Conversely, FLS can also respond to pro-inflammatory factors secreted by MLS, such as TNF-alpha and IL-1. This interaction between FLS and MLS synergistically promotes the role of FLS in the RA synovial microenvironment. Thus, the aggressive contribution of FLS to the RA pathology is closely associated with MLS and the dual roles (“passive responder” and “imprinted aggressor”) of FLS in RA warrant further investigation, especially with regard to their interplay with MLS.

### MLS in RA

**Figure 3:**
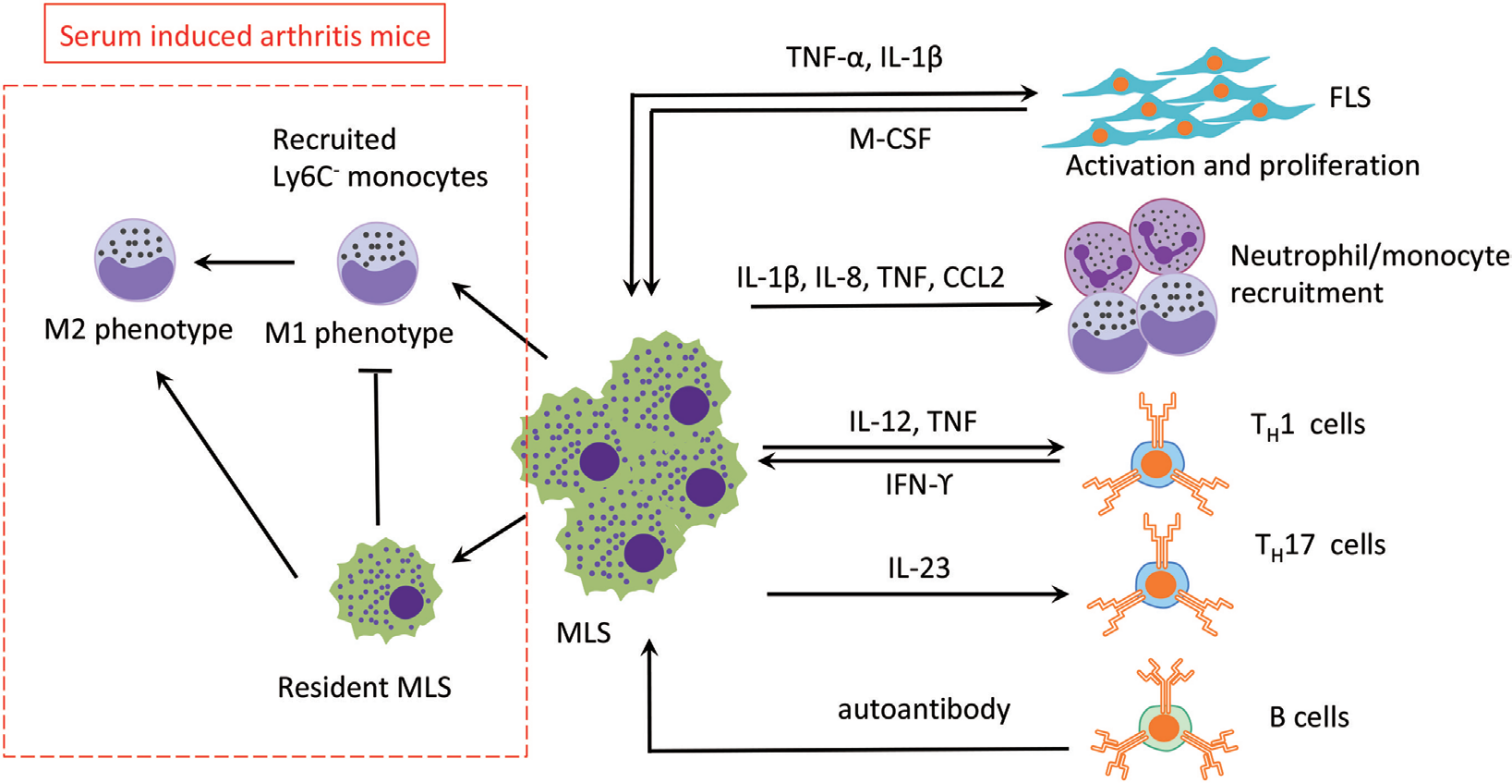
The roles of MLS in RA. The three main effects of MLS in RA are mediated by cytokines secretion: (1) FLS activation, (2) recruitment of neutrophil/monoctes, and (3) T cells polarization. MLS are also affected by direct cell contact or indirect cytokine production by FLS, T cells, and B cells (autoantibody). In addition, in a serum-induced arthritis mouse, circulation recruited Ly6C^−^ monocytes limits development of arthritis mice and promotes the switch from M1 to M2 in the arthritis joint. Abbreviations: TNF, tumor necrosis factor; IL-1β, interleukin 1β; IL-8, interleukin 8; IL-23, interleukin 23; M-CSF, macrophage colony-stimulating factor; IFN- γ, interferon γ; CCL2, monocyte chemoattractant protein 1; T_H_1, type 1T helper cells; T_H_17, type 17 helper cells; MSL, macrophage-like synoviocytes; FSL, fibroblast-like synoviocytes; RA, rheumatoid arthritis.

Compared with FLS, the role of MLS in RA is underinvestigated, probably due to the limited number of cells *in vivo* and their slow or non-existent proliferation *in vitro*. It has been shown that MLS contribute to RA progression by secretion of various factors, including reactive oxygen species, nitric oxide intermediates, and matrix-degrading enzymes (40). In addition, MLS produce different kinds of cytokines in the rheumatoid synovium that can accelerate inflammation by recruiting other immune cells and activating FLS (41). Therefore, it would be beneficial if we could target pro-inflammatory TNF-α selectively and spare of anti-inflammatory IL-10 in therapy. This notion is supported by the observation that resident MLS limit the development of arthritis in a mouse model by inhibit recruitment of inflammatory Ly6C^−^ monocytes and promote the switch from M1 to M2 in the arthritic joint (24). In an unexpected observation, it could be demonstrated that Ly6C^−^ monocytes are recruited to the inflamed synovium and differentiate into inflammatory M1 macrophages in the initiation and progression stage of arthritis. During the development of arthritis, resident MLS prevent Ly6C^−^ monocytes recruitment and induce these inflammatory M1 macrophages to polarize toward the alternatively activated M2 phenotype, which leading to the resolution of joint inflammation. The role of MLS in the RA synovium has been summarized in Figure 3 below.

### The Interaction Between MLS and FLS in RA

Due to the roles of MLS/FLS in RA synovial tissue, the interaction of these two cellular components is critical for the initiation of inflammation and the subsequent damage to the joint in RA. The interaction of FLS and MLS induces the secretion of a panel of cytokines, including IL-6, IL-8, and GM-CSF. The treatment of the inflammation with anti-inflammatory cytokines (such as IL-4, IL-10, IL-13, or IL-1 receptor antagonist) suppresses the secretion of pro-inflammatory cytokines and ameliorates the inflammatory response (42). In addition, in an *in vitro* co-culture of monocytes and FLS, a neutralization of the CD14 molecule also suppressed the production of pro-inflammatory cytokines (43). Furthermore, co-culture models of mouse FLS and MLS *in vitro* resulted in an activation that induced cartilage damage (44). This result has been validated in *in vitro* co-cultures of purified human FLS and myelomonocytic cells (45).

**Figure 4:**
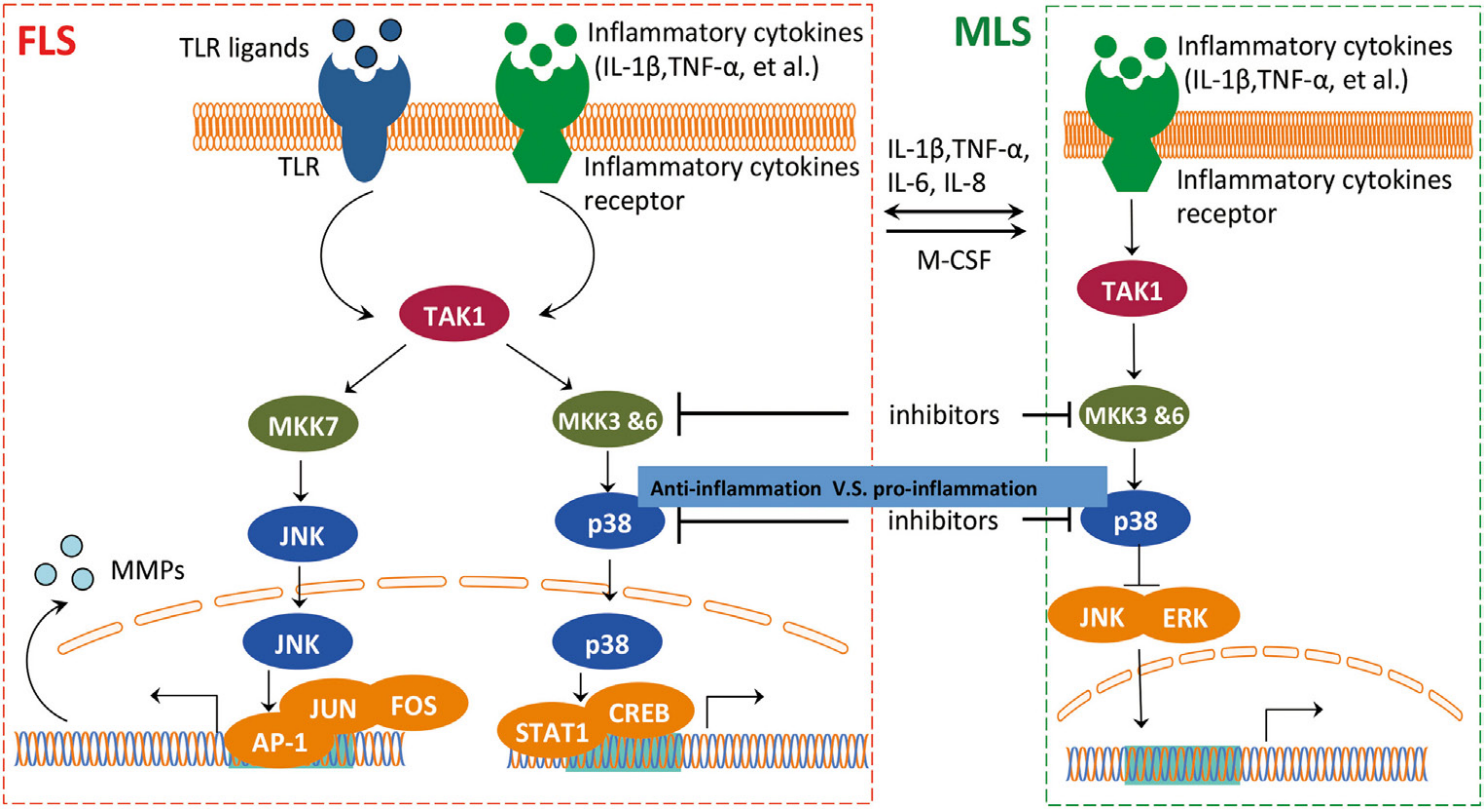
The interaction between FLS and MLS in RA. FLS and MLS cross talk through secreted cytokines and MAPK pathway. FLS and MLS mutually activate *via* cytokines production; in addition, the pro-inflammatory and anti-inflammatory contest highlights the important role of the interaction between MLS and FLS *via* MAPK pathway in RA. Abbreviations: MAPK, mitogen-activated protein kinase; TAK1, TGF-beta activated kinase 1; MKK3, MAP kinase kinase 3; MKK6, MAP kinase kinase 6; MKK7, MAP kinase kinase 7; JNK, c-Jun N-terminal kinases; AP-1, APETALA 1; FOS, FBJ murine osteosarcoma viral oncogene homolog; STAT1, signal transducer and activator of transcription 1; CREB, cAMP responsive element binding protein 1; ERK, extracellular signal-regulated kinase; MSL, macrophage-like synoviocytes; FSL, fibroblast-like synoviocytes; RA, rheumatoid arthritis.

The cross talk of FLS and MLS is mediated through the mitogen-activated protein kinase (MAPK) pathway (46) (Figure 4). It is well documented that the MAPK pathway is positively correlated with an aggressive behavior of FLS in RA (47, 48). However, p38 MAPK inhibitors do not exert a therapeutic effect on RA (49), probably due to a competition between the pro-inflammatory and anti-inflammatory activities of MLS and FLS within the RA synovium. A p38 inhibitor can induce activation of pro-inflammatory pathways in MLS, and this pro-inflammatory effect could overpower the anti-inflammatory effects of p38 MAPK inhibition in FLS. This speculative contest highlights the need for a more detailed analysis of the interaction between MLS and FLS in RA. Inhibiting p38 in RA that is dominated by MLS cytokines could paradoxically suppress anti-inflammatory functions and interfere with therapy efficacy. Targeting upstream kinases MKK3 or MKK6 that regulate p38 could be more effective by suppressing pro-inflammatory cytokines while preventing a decrease in the expression of the anti-inflammatory IL-10 in MLS. Therefore, blocking MKK3 or MKK6 shows promising therapeutic efficacy *in vivo* in mouse models, indicating the pharmacological potential of MKK3 and MKK6 inhibitors for MLS/FLS-targeted therapy of RA (50, 51).

## The Development of Anti-Arthritis Drugs Targeting MLS and FLS

### Targeting of Macrophages-Secreted Pro-Inflammatory Cytokines

Disease-modifying anti-rheumatic drugs (DMARDs) are the most commonly used drugs for RA (52). Although drugs are not specifically designed to target MLS, DMARDs exert their effect *via* regulation of macrophage-secreted pro-inflammatory cytokines, in particular, IL-6 and TNF-α. These pro-inflammatory cytokines have been extensively studied in clinical trials, and drugs that specifically target these two cytokines have been identified and are used in therapy (53). TNF monoclonal antibodies (including infliximab, adalimumab, and golimumab) and TNF receptor 2-IgG1 fusion protein (including etanercept) are two types of anti-TNF drugs that cause a transient TNF deficiency (54). The current underlying assumption about the mechanism how those drugs work is that pro-inflammatory effects of TNF are blocked by neutralizing the cytokine. However, recently published data argue that when TNF is reduced, type-2 functions increase (55). Therefore, anti-TNF drugs may work in multiple layers to regulate inflammation which could explain the high rate of therapy failure. Second, a panel of IL-6-targeting agents, such as Siltuximab (chimeric anti-IL-6 Ab) (56), Sirukumab (fully human anti-IL-6 Ab) (57), Clazakizumab (humanized anti-IL-6 Ab) (58), Olokizumab (humanized anti-IL-6 Ab) (59), Sarilumab (fully human anti-IL-6R Ab) (60), and tocilizumab (61), are in different stages of clinical trials and are promising or have been approved for clinical RA treatment as replacement in a case of anti-TNF therapy failure (62).

### Targeting of FLS in RA

Based on previous findings, targeting of FLS definitely should be considered an option for RA therapy. FLS are directly involved in RA pathogenesis and produce pro-inflammatory cytokines. Therefore, either alternative or complementary anti-inflammatory or anti-immune therapy can be used to target FLS. CDH11 is the first specific factor proven to be an essential, functional surface protein of FLS in RA. As indicated in Table 1, the expression of CDH11 by FLS is cell-specific and not detectable on other immune cells in RA. Moreover, an anti-CDH11 antibody is currently under investigation for RA treatment (63). Anti-CDH11 in FLS could reduce secretion of pro-inflammatory cytokines IL-6 and consequently synergize with TNF-α and IL-1β in the induction of IL-6 *via* repressing MAPK and NF-κB pathways.

**Table 1:** The specific markers for fibroblast-like synoviocytes and macrophage-like synoviocytes in mice.

Fibroblast-like synoviocytes		Macrophages-like synoviocytes	
**Common proteins**			
MHC class II, major histocompatibility complex			

**Surface proteins**			
CD55	DAF, decay accelerating factor	CD11b/c	Integrin adhesion molecular and complement receptor

CD90	Thy-1	CD14	LPS/LBP receptor

ICAM-1	Intercellular adhesion molecular-1	CD16	Immunoglobulin G Fc receptor

Vascular cell adhesion molecule-1	CD106, vascular factor adhesion molecular-1	CD45	Leukocyte common antigen

Cadherin-11	Calcium-dependent adhesion factor-11	CD68	Lysosomal glycoprotein

**Intracellular proteins**			
UDPGDH	Uridine diphosphoglucose-dehydrogenase		

Type IV collagen	Structural proteins		

Type V collagen	Structural proteins		

Vimentin	Intermediate filament		

Besides the specific expression of CDH11 in FLS, other common markers of both FLS and MLS can also be considered as potential RA targets. For example, suppression of an essential gene, such as the c-Jun N-terminal kinase (JNK) in the MAPK pathway also attenuates the effect of RA in rodent models (64). The JNK inhibitor SP600125 (anthra[1,9-cd]pyrazol-6(2H)-one) inhibited IL-1-induced accumulation of phospho-Jun and induction of c-Jun transcription in FLS and decreased the joint inflammation in rat adjuvant-induced arthritis. Due to the common expression in both FLS and MLS, targeting JNK1 will block its endogenous expression in both synovial cell types (65, 66). Furthermore, it has been suggested that FLS and MLS cooperate in a synergistic manner in the inflamed synovium. Moreover, mice in an arthritis model which had been injected with MKK7 anti-sense oligonucleotides had significantly less severe arthritis by reducing phospho-JNK and phospho-c-Jun-mediated inflammation in joint synoviocytes. This effect suggests that MKK7 is also a potential target in human RA but needs further validation (67).

### Targeting of MLS in RA

Given the essential role of MLS in driving inflammation in the RA synovium, deletion of inflammatory MLS is a potential treatment for RA. This idea has been validated by using an immunotoxin directed to CD64 which is a marker of MLS in RA. This experimental therapy selectively eliminated macrophages in RA. It was demonstrated that selective elimination of MLS in RA relieved the pathological phenotype in RA synovial tissue explants *in vitro* (68). In addition, a follow-up study showed that an *in vivo* depletion of CD64^+^ macrophages in a rat arthritis model inhibited the joint inflammation in adjuvant-induced arthritis (69). The treatment with CD64-targeting immunotoxin resulted in a substantial reduction in macrophage numbers and improved inflammatory conditions (69). These experiments that targeted CD64^+^ macrophages indicate that targeting MLS may be a useful clinical strategy.

## Other MLS- or FLS-Associated Potential RA Therapies

### Targeting of Monocytes

Recruitment of monocytes, the precursors of mature inflammatory macrophages, into affected joints is essential for an initiation and progression of joint inflammation. Therefore, several methods have been established to remove circulating monocytes or prevent the recruitment of monocytes into joints, including use of immune-modifying microparticles (polystyrene, micro-diamonds, or biodegradable poly-microparticles), either in experimental or therapeutic applications (70, 71). As expected, arthritis was suppressed in mouse models. However, as mentioned above, receptor redundancy is a bottleneck in monocyte-targeting therapy for RA. Due to the presence of redundant receptors that recruit monocytes from the circulation during RA progression (72), human clinical trials with anti-CCR2 antibodies have ended in failure (73). Approved in 2015 for the treatment of liposarcoma and leiomyosarcoma, the chemotherapeutic anti-CCR2 agent trabectedin can selectively deplete monocytes and macrophages in blood and tissues (74) and could be worth testing in RA despite its potentially serious off-target effects, given that other antitumor drugs (such as methotrexate) have been proven to be useful in chronic autoimmune inflammations such as RA.

### GM-CSF/GM-CSF-R Pathway

Targeting of GM-CSF and GM-CSF-R is under development for RA therapy in both preclinical studies and clinical trials (75). Blocking CSF-1 can relieve arthritis in an experimental RA mouse model (76). Increased GM-CSF-R^+^ MLS were observed in synovia from RA patients, and experimental therapy with an anti-GM-CSF-R antibody (77) showed inflammation-reducing effects on inflammatory MLS in the collagen-induced arthritis mouse model. In addition, the GM-CSF-R antagonist mavrilimumab has already shown a therapeutic effect in patients with RA in phase IIa trials (78). A second phase IIb study also showed promising results in 2017 (79).

Interferon regulatory factor 5 (IRF5) is a downstream target of GM-CSF/GM-CSF-R signaling in monocytes (80). An increased number of MHCII^+^ MLS is suppressed in joints of IRF5 knockout mice in a mouse RA model (81). IRF5 can also promote differentiation of monocytes into CD64^+^ macrophages in the inflamed joint (81). In addition, the recruitment of IRF5^+^ monocytes into the arthritic joint is blocked in CCR2^−/−^ mice (81), confirming that IRF5 is involved in GM-CSF signaling (82). Therefore, IRF5 is a promising potential therapeutic target. Suppression of IRF5 by siRNA *in vivo* reprograms the macrophage phenotype, reverses inflammation, and accelerates healing of cutaneous and myocardial lesions (83). However, the off-target use of siRNA is an inevitable question, and its safety, efficacy, and off-target effect has to be evaluated critically.

## Future Perspective

Even in a “homogeneous” cell population (such as MLS and FLS), the isolation of specific cellular groups is dependent on positive or negative sorting according to several markers. Therefore, a considerable variation between individual cells will still be detected. Detection of variations at the single-cell level can reveal the heterogeneity of FLS and MLS involvement in the RA synovium from individual patients, enabling identification of cell origin and more specific cell populations for the use in RA therapy. For example, single-cell RNA sequencing provides unbiased identification of FLS and MLS subtypes in RA synovium (84). Variable cellular states and subsets of FLS and MLS can be stratified using single-cell transcriptome sequencing, enabling a detailed understanding of cellular heterogeneity (85). In addition, an analysis of the proteome needs to be performed in MLS and FLS (86). Single-cell proteomic data are important for a detailed understanding of cellular heterogeneity at the post-translational level, since the correlation between mRNA and protein is not always strong enough. Single-cell epigenomic analysis using state-of-the-art techniques such as CHiP seq is another cutting-edge technology that can identify the variation in different epigenetic modifications (including RNA, DNA, and histone modifications), providing a detailed and novel method to elucidate the mechanisms of RA pathogenesis (87). Another advantage is that single-cell assays require small amounts of biological material (88). Therefore, adequate sampling of RA synovium is no longer a major obstacle.

## Conclusion

**Figure 5:**
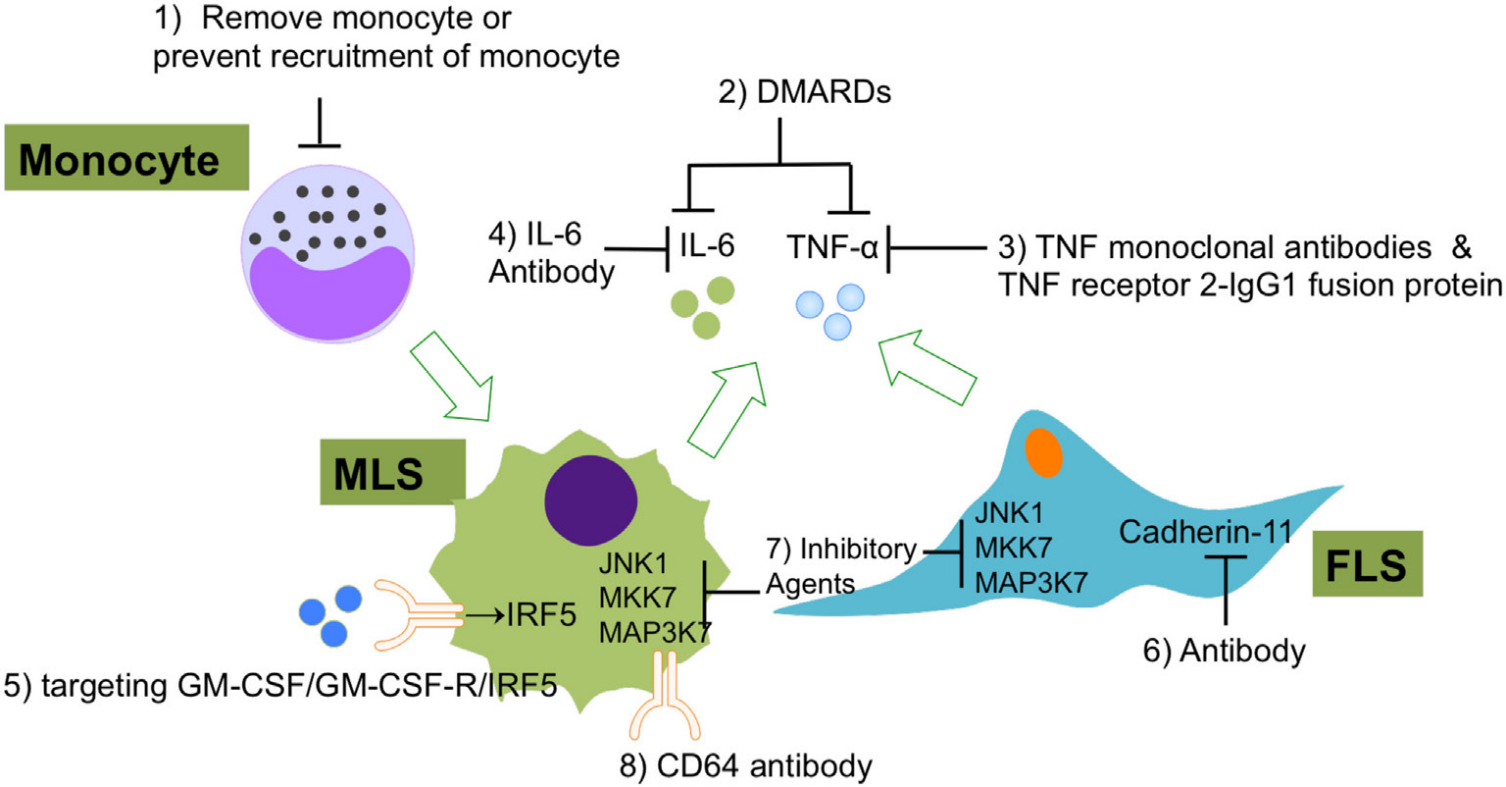
The current experimental and clinical FLS- and MLS-targeting treatments of RA. (1) Using of immune-modifying microparticles (polystyrene, micro-diamonds, or biodegradable poly-microparticles) to remove circulating monocytes or prevent the recruitment of monocytes into joints; (2) DMARDs to inhibit FLS or MLS secreted pro-inflammatory cytokines; (3) TNF-α monoclonal antibodies and TNF receptor 2–IgG1 fusion protein to specifically repress TNF-α-induced inflammation; (4) IL-6 antibodies to specifically repress IL-6-induced inflammation; (5) agents that targeting GM-CSF/GM-CSF-R/IRF-5 regulatory axis in MLS; (6) CDH11 antibody (FLS); (7) inhibitory agents to target JNK1, MKK7, and MAP3K7 in FLS and MLS; (8) CD64 antibody (FLS). Abbreviations: DMARDs, disease-modifying anti-rheumatic drugs; CD64, complement component 64; RA, rheumatoid arthritis; FLS, fibroblast-like synoviocytes; MSL, macrophage-like synoviocytes; CDH11, cadherin-11.

Although the main two types of synovicytes in synovium (MLS and FLS) have been identified for a long time, researcher began to notice the essential roles of these two types of cells in the RA development only recently. MLS and FLS have been proven to contribute to RA pathology collaboratively. A range of novel methods have been developed for the evaluation of new potential RA therapies that target MLS and FLS in the inflamed synovium (Figure 5). Some current therapeutic agents for RA can block RA inflammation by suppressing pathological MLS and FLS functions. However, the effectiveness and safety of these potential therapies must be compared with those of current standard RA treatments, such as biologic agents, non-steroidal anti-inflammatory drugs, and DMARDs. It has been well understood that MLS and FLS are involved in the entire pathological process of RA but now the specific roles of these two cellular components at different stages of RA are coming into focus. However, so far there is no specific RA therapy that only targets MLS or FLS. As mentioned above, the origins of MLS and FLS are still elusive. Furthermore, the heterogeneity of the MLS and FLS response *in vivo* in RA has not yet been analyzed at the single-cell level. Finally, to understand the cross talk between microenvironment and MLS/FLS in synovium, it will be essential to deciper the contribution of this cell types to RA pathology. We suggest that the abovementioned options should be considered and deserve further investigation in future work.

## Author Contributions

JT drafted the manuscript. WH, PZ, XW, HK, and WW revised the manuscript.

## Conflict of Interest Statement

The authors declare that the research was conducted in the absence of any commercial or financial relationships that could be construed as a potential conflict of interest.
